# Dietary Omega-3 Fatty Acids Suppress Experimental Autoimmune Uveitis in Association with Inhibition of Th1 and Th17 Cell Function

**DOI:** 10.1371/journal.pone.0138241

**Published:** 2015-09-22

**Authors:** Hiromi Shoda, Ryoji Yanai, Takeru Yoshimura, Tomohiko Nagai, Kazuhiro Kimura, Lucia Sobrin, Kip M. Connor, Yukimi Sakoda, Koji Tamada, Tsunehiko Ikeda, Koh-Hei Sonoda

**Affiliations:** 1 Department of Ophthalmology, Yamaguchi University Graduate School of Medicine, Ube, Yamaguchi, Japan; 2 Angiogenesis Laboratory, Department of Ophthalmology, Massachusetts Eye and Ear Infirmary, Harvard Medical School, Cambridge, Massachusetts, United States of America; 3 Department of Immunology, Yamaguchi University Graduate School of Medicine, Ube, Yamaguchi, Japan; 4 Department of Ophthalmology, Osaka Medical Collage, Osaka, Japan; Max Delbrueck Center for Molecular Medicine, GERMANY

## Abstract

Omega (ω)–3 long-chain polyunsaturated fatty acids (LCPUFAs) inhibit the production of inflammatory mediators and thereby contribute to the regulation of inflammation. Experimental autoimmune uveitis (EAU) is a well-established animal model of autoimmune retinal inflammation. To investigate the potential effects of dietary intake of ω-3 LCPUFAs on uveitis, we examined the anti-inflammatory properties of these molecules in comparison with ω-6 LCPUFAs in a mouse EAU model. C57BL/6 mice were fed a diet containing ω-3 LCPUFAs or ω-6 LCPUFAs for 2 weeks before as well as after the induction of EAU by subcutaneous injection of a fragment of human interphotoreceptor retinoid-binding protein emulsified with complete Freund’s adjuvant. Both clinical and histological scores for uveitis were smaller for mice fed ω-3 LCPUFAs than for those fed ω-6 LCPUFAs. The concentrations of the T helper 1 (Th1) cytokine interferon-γ and the Th17 cytokine interleukin-17 in intraocular fluid as well as the production of these cytokines by lymph node cells were reduced for mice fed ω-3 LCPUFAs. Furthermore, the amounts of mRNAs for the Th1- and Th17-related transcription factors T-bet and RORγt, respectively, were reduced both in the retina and in lymph node cells of mice fed ω-3 LCPUFAs. Our results thus show that a diet enriched in ω-3 LCPUFAs suppressed uveitis in mice in association with inhibition of Th1 and Th17 cell function.

## Introduction

Lipid autacoids have well-established roles in physiology and pathophysiology. Omega (ω)–3 and ω-6 long-chain polyunsaturated fatty acids (LCPUFAs) are two classes of dietary lipid [1] that are highly enriched in the retina [2] and which have opposing physiological effects. Mammals depend on dietary intake of LCPUFAs because they lack the enzymes capable of synthesizing these molecules de novo, with ω-6 LCPUFAs being the primary polyunsaturated fatty acids present in Western diets and ω-3 LCPUFAs serving as substrates for the generation of potent and protective autacoids such as resolvins and neuroprotectin D1 [3]. The balance of eicosanoids derived from ω-3 and ω-6 LCPUFAs is an important determinant of cardiovascular [4–6] and renal [7] function, vascular tone [8], and inflammatory and immune processes [9]. ω-3 LCPUFAs have been shown to suppress ocular inflammation in a mouse endotoxin-induced uveitis (EIU) model [10] as well as ocular neovascularization in animal models of age-related macular degeneration [11, 12] or of oxygen-induced retinopathy [3]. The mechanism of such suppression of ocular inflammation by ω-3 LCPUFAs has remained unclear, however.

Uveitis is a sight-threatening intraocular inflammatory disease [13–16]. Experimental autoimmune uveitis (EAU) is an animal model of uveitis that shares many pathological features with human uveitis [17–21]. EAU is induced by injection of animals with purified retinal antigens such as S-Ag, human interphotoreceptor retinoid-binding protein (hIRBP), rhodopsin (or opsin), phosducin, or recoverin. It has been shown to be mediated by T helper 1 (Th1) and Th17 cells [22–24], which are generated from naive T cells in response to exposure to pro-inflammatory cytokines. The effects of dietary intake of LCPUFAs on local inflammation associated with uveitis are unknown. The purpose of this study is to investigate the possible anti-inflammatory effects of dietary intake of ω-3 LCPUFAs in a mouse EAU model.

## Materials and Methods

### Animals and ω-3 or ω-6 LCPUFA diets

Six-week-old female C57BL/6 mice were obtained from Chiyoda Kaihatsu (Tokyo, Japan) and were fed a diet containing ω-3 or ω-6 LCPUFAs beginning 2 weeks before the induction of EAU. The completely defined isocaloric diets were enriched with 2% total fatty acids consisting of either ω-3 LCPUFAs (1% docosahexaenoic acid [DHA] and 1% eicosapentaenoic acid [EPA]) or ω-6 LCPUFAs (2% arachidonic acid [AA]) (Oriental Yeast, Tokyo, Japan). A separate group of mice was maintained on a control chow. Mice were maintained in individual cages at 22° to 24°C and a relative humidity of 50% to 70% and with a 12-h-light, 12-h-dark cycle (lights on from 0700 to 1900 hours). The condition of the animals was monitored at least once a day and at each anesthetization for scoring of EAU (see below). They were killed by cervical dislocation at 21 or 28 days after induction of EAU. Animals were treated in accordance with the ARVO Statement for the Use of Animals in Ophthalmic and Vision Research, and the study was approved by the Animal Care and Use Committee of Yamaguchi University.

### EAU model

EAU was induced in mice at 8 weeks of age by subcutaneous injection of 50 μg of an NH_2_-terminal peptide fragment (residues 1–20, GPTHLFQPSLVLDMAKVLLD) of hIRBP (Scrum, Tokyo, Japan) emulsified with complete Freund’s adjuvant containing *Mycobacterium tuberculosis* H37Ra at 6 mg/ml (Difco, Detroit, MI, USA). The emulsion (total of 100 μl) was injected into a footpad and the inguinal region. Pertussis toxin (100 μg) (Sigma, St. Louis, MO, USA) was also injected intraperitoneally at the same time.

### Scoring of EAU

At 7, 14, 21, and 28 days after induction of EAU, mice were anesthetized by intraperitoneal injection of a mixture of ketamine (90 mg/kg) (Daiichi Sankyo, Tokyo, Japan) and xylazine (10 mg/kg) (Bayer Yakuhin, Osaka, Japan) and their pupils were dilated by topical instillation of 0.5% tropicamide and 0.5% phenylephrine hydrochloride ophthalmic solutions (Santen, Osaka, Japan). Eye gel (Scopisol; Senju, Osaka, Japan) was applied to the cornea for contact with a digital medical scope (VersaCam; NIDEK, Aichi, Japan), focus and illumination were adjusted, and images of the fundus were obtained. Clinical score for EAU was graded on the basis of the photographs by retinal specialists in a blinded manner on a scale of 0 to 5: 0, no evidence of inflammation; 1, focal vasculitis and/or spotted soft exudate of less than 5 spots; 2, linear vasculitis and/or spotted soft exudate within half of the retina; 3, linear vasculitis and/or spotted soft exudate over half of the retina; 4, retinal hemorrhage along with severe vasculitis and/or spotted soft exudate; 5, exudative retinal detachment or subretinal hemorrhage.

For histological assessment, mice were killed by cervical dislocation 21 days after induction of EAU. The eyeballs were removed and fixed in 4% paraformaldehyde for preparation of paraffin-embedded sections (5 μm) in the papillary—optic nerve plane. The sections were stained with hematoxylin-eosin. EAU was scored in each eye on a scale of 0 to 4: 0, no disease and normal retinal architecture; 0.5, mild inflammatory cell infiltration and no tissue damage; 1, mild infiltration in the uvea, vitreous, and retina as well as the presence of retinal folds, vasculitis, and one small granuloma; 2, moderate infiltration in the uvea, vitreous, and retina as well as the presence of retinal folds, vasculitis, focal shallow detachments, small granulomas, and focal photoreceptor cell damage; 3, moderate to severe infiltration in the uvea, vitreous, and retina as well as the presence of extensive retinal folding with large detachments, subretinal neovascularization, moderate photoreceptor cell damage, and medium-size granulomatous lesions; 4, severe infiltration, diffuse retinal detachment, subretinal neovascularization and hemorrhage, extensive photoreceptor cell damage, and large granulomatous lesions [21].

### Analysis of fatty acids in serum

Retro-orbital sinus blood was collected rapidly after removal of the eyeballs from anesthetized mice at 14 days after induction of EAU. The blood samples were collected into tubes under nitrogen, allowed to stand for 1 h in room air, and then centrifuged at 845 × *g* for 15 min at 4°C. The serum supernatant was stored at –80°C until analysis of fatty acid composition of lipids by gas chromatography at SRL Clinical Laboratory (Tokyo, Japan).

### Analysis of cytokines and chemokines in intraocular fluid

Mice were killed and the eyeballs were removed 14 days after EAU induction. Each eyeball was trimmed of extraocular muscles and soft tissue and then crushed with the use of a BioMasher (Sarstedt, Germany). The crushed sample was centrifuged at 9000 × *g* for 30 s, and the resulting supernatant (intraocular fluid) was stored at –80°C until assay of cytokine and chemokine concentrations with the use of a Bio-Plex Pro Mouse Cytokine 23-Plex Panel and Bio-Plex Manager software version 4.1.1 (Bio-Rad, Hercules, CA, USA).

### Isolation of RNA and RT

Retinal tissue isolated from the eye 21 days after EAU induction was added to RNAlater solution (Life Technologies, Carlsbad, CA, USA) for RNA stabilization. Total RNA was then isolated from the retinal tissue as well as from purified CD4^+^ T cells with the use of an RNeasy Mini Kit (Qiagen, Venlo, the Netherlands) and was subjected to reverse transcription (RT) with the use of a SuperScript III kit (Invitrogen, Carlsbad, CA, USA). The resulting cDNA samples were stored at −80°C until analysis.

### Isolation and stimulation of CD4^+^ T cells

Mice were killed 14 days after EAU induction, and the superficial cervical, axillary, brachial, and inguinal lymph nodes were collected for isolation of CD4^+^ T cells by magnetic-activated cell sorting (MACS) with the use of a kit (Miltenyi Biotec, Palo Alto, CA, USA). Feeder cells (5 × 10^7^) prepared from the spleen of normal mice were incubated with mitomycin C (50 μg/ml) in phosphate-buffered saline for 20 min at 37°C and then washed three times with RPMI 1640 medium supplemented with 2% penicillin-streptomycin and 10% fetal bovine serum. Isolated CD4^+^ T cells (2 × 10^5^ in 50 μl per well of a 96-well plate) and mitomycin C—treated feeder cells (1 × 10^6^ in 100 μl) were cultured together for 48 h at 37°C in RPMI 1640 and in the absence or presence of hIRBP(1–20) (10 μg/ml). The culture supernatants were then collected and stored at –80°C until assay of cytokine concentrations with enzyme-linked immunosorbent assay (ELISA) kits (R&D Systems, Minneapolis, MN, USA).

### Real-Time PCR analysis

Real-time polymerase chain reaction (PCR) analysis of retinal or CD4^+^ T cell cDNA samples was performed with the use of a StepOne Real-Time PCR System (Applied Biosystems, Foster City, CA, USA) and mouse TaqMan gene expression assays (Applied Biosystems) for Tbx21 (T-bet, Mm00450960_m1) and Rorc (RORγt, Mm01261022_m1). Data were normalized by the corresponding abundance of β-actin cDNA. Furthermore, we prepared samples for RT-PCR analysis at 21 days after EAU induction.

### Statistical analysis

Quantitative data are presented as means ± SD and were analyzed with the use of Prism version 6 for Windows (GraphPad Software, San Diego, CA, USA). EAU scores were compared among groups with the nonparametric Mann-Whitney *U* test or Tukey-Kramer test. Other data were compared between groups with the unpaired Student's *t* test. A *P* value of <0.05 was considered statistically significant.

## Results

### Clinical score

**Fig 1:**
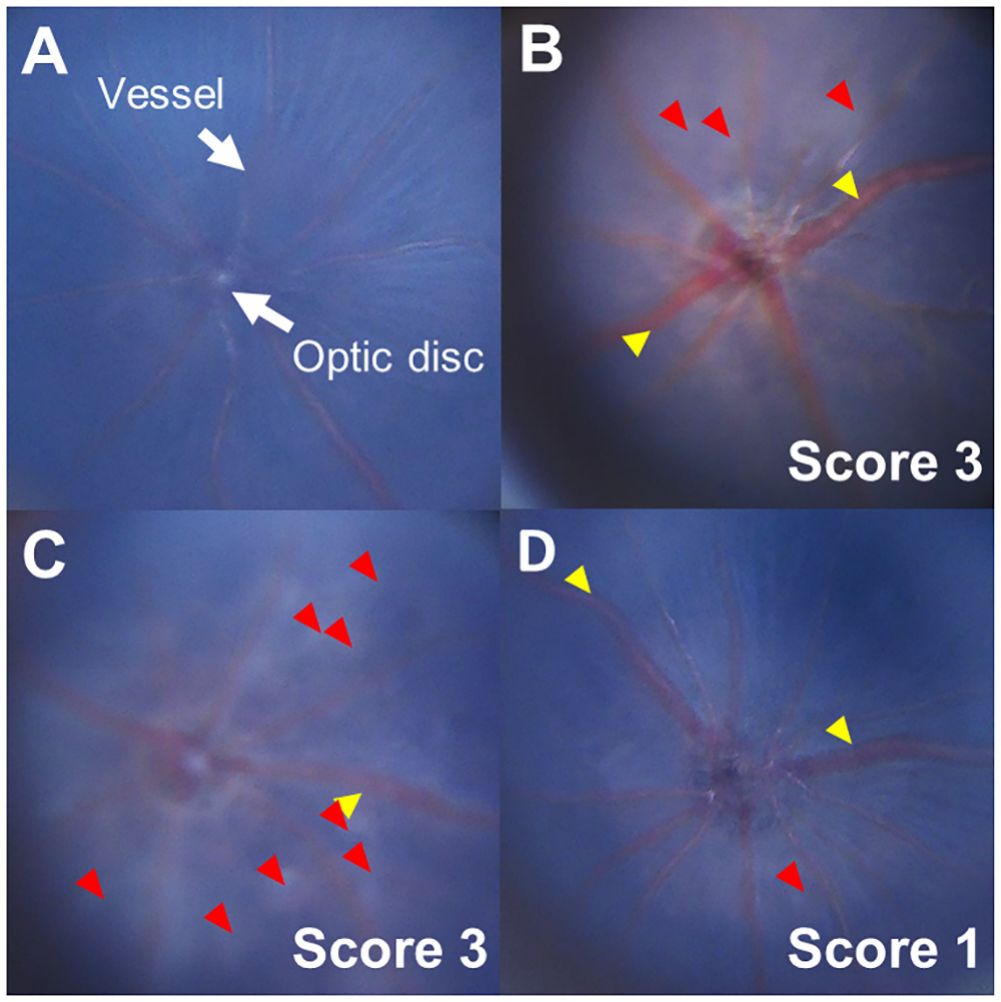
Representative fundus findings for mice with EAU. Mice were anesthetized for examination with a digital medical scope at 14 days after induction of EAU. Fundus images are shown for naive mice (A) as well as for those injected with hIRBP(1–20) and maintained on a control (B), ω-6 LCPUFA (C), or ω-3 LCPUFA (D) diet. The clinical score for EAU determined from the fundus photographs is indicated. Yellow arrowheads, retinal vasculitis with cuffing; red arrowheads, retinal exudate.

**Fig 2:**
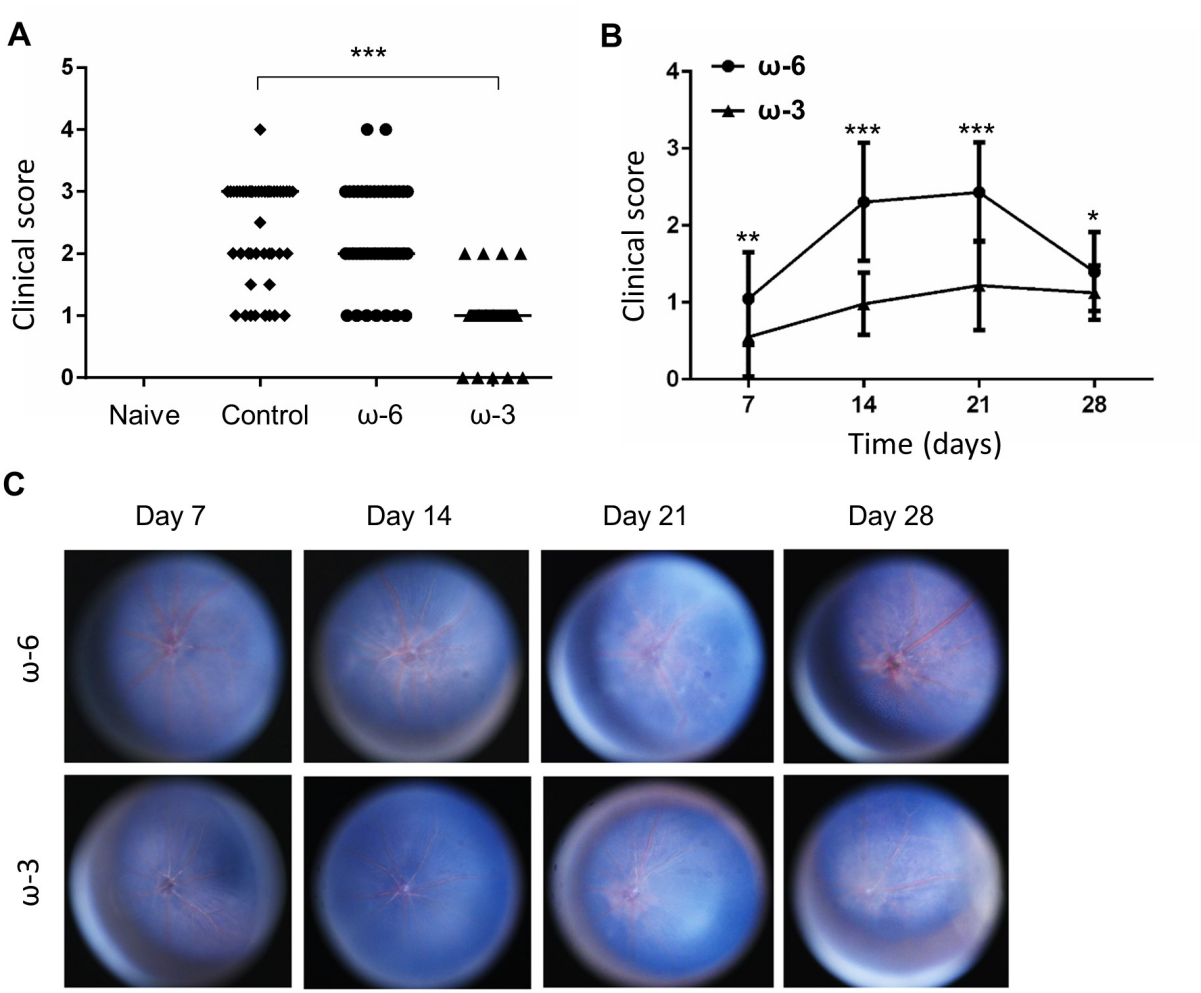
Clinical score for EAU. (A) EAU clinical score determined from fundus images obtained from mice at14 days after injection with hIRBP(1–20) and maintenance on a control (*n* = 36), ω-6 LCPUFA (*n* = 49), or ω-3 LCPUFA (*n* = 56) diet. Naive mice (*n* = 10) were similarly examined. ***P < 0.001. (B) EAU clinical score for mice in the ω-6 and ω-3 groups at 7, 14, 21, and 28 days after EAU induction. Data are means ± SD. ***P < 0.001, **P < 0.01, *P < 0.05. (C) Representative fundus photographs for mice in the ω-6 and ω-3 groups at 7, 14, 21, and 28 days after EAU induction.

Examination of the fundus of naive mice revealed a normal optic disk and healthy vessels, with no vasculitis or exudates (Fig 1A). Whereas the fundus of mice at 14 days after injection with hIRBP(1–20) and maintenance on a control (Fig 1B) or ω-6 LCPUFA (Fig 1C) diet manifested both linear vasculitis and exudates, that of mice fed an ω-3 LCPUFA diet (Fig 1D) appeared similar to that of naive mice. Quantitative analysis (Fig 2A) showed that the clinical score of the ω-3 group (0.98 ± 0.40, mean ± SD) was significantly reduced compared with that of the ω-6 group (2.4 ± 0.79) or the control diet group (2.3 ± 0.77). Examination of the fundus at various times after the induction of EAU revealed that the clinical score for the ω-3 group was smaller than that for the ω-6 group at 14 and 21 days (Fig 2B and 2C). These results thus suggested that dietary intake of ω-3 LCPUFAs suppressed the inflammation associated with EAU.

### Histological score

**Fig 3:**
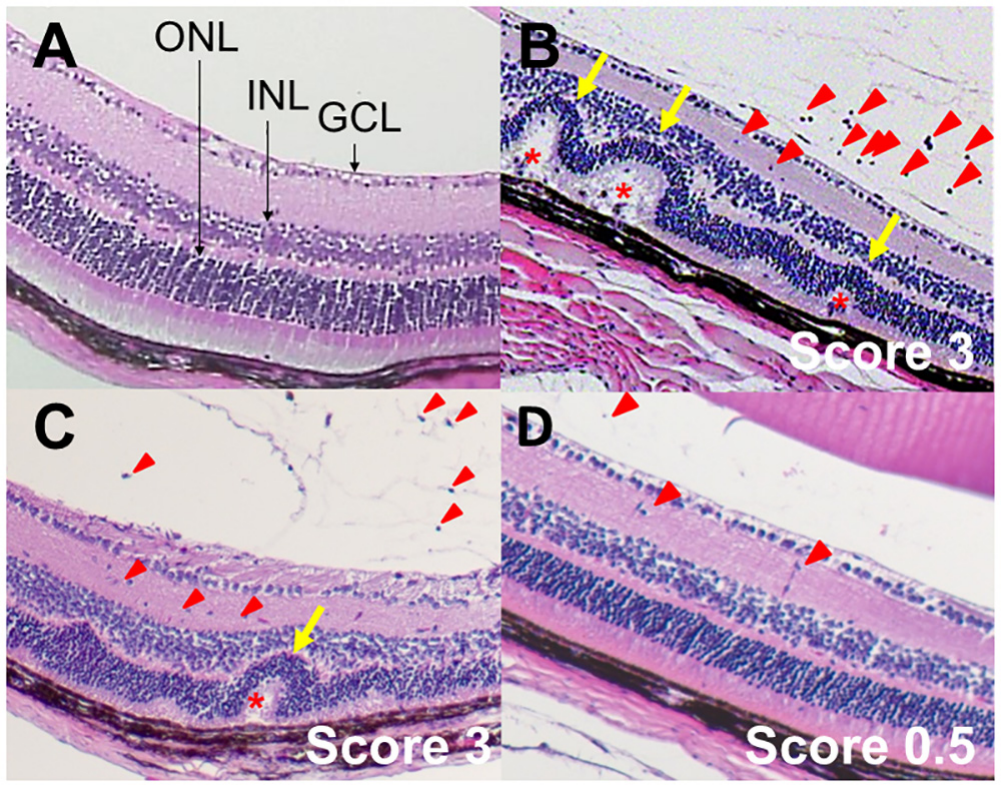
Representative histological findings for EAU mice. Sections of the eye from naive mice (A) as well as from EAU mice at 21 days after injection with hIRBP(1–20) and maintenance on a control (B), ω-6 LCPUFA (C), or ω-3 LCPUFA (D) diet were stained with hematoxylin-eosin. The histological score for EAU determined from the histological findings is indicated. GCL, ganglion cell layer; INL, inner nuclear layer; ONL, outer nuclear layer. Red arrowheads, inflammatory cells in the vitreous and retina; yellow arrows, retinal folds; red asterisks, granulomatous lesions.

To confirm the anti-inflammatory effect of ω-3 LCPUFAs in EAU mice, we performed histological analysis at 21 days after hIRBP(1–20) injection. Compared with the normal appearance of histological sections prepared from the eye of naive mice (Fig 3A), inflammatory cell infiltration and granulomatous lesions were detected in those from mice injected with hIRBP(1–20) and fed either the control (Fig 3B) or ω-6 LCPUFA (Fig 3C) diet. In contrast, these inflammatory changes were greatly attenuated in mice fed the ω-3 LCPUFA diet (Fig 3D). The histological scores were 0, 3, 3, and 0.5 for mice in the naive, control diet, ω-6, and ω-3 groups, respectively. These results thus provided further support for the notion that dietary intake of ω-3 LCPUFAs ameliorated retinal inflammation in EAU mice.

### Inflammatory cytokines and chemokines in intraocular fluid

**Fig 4:**
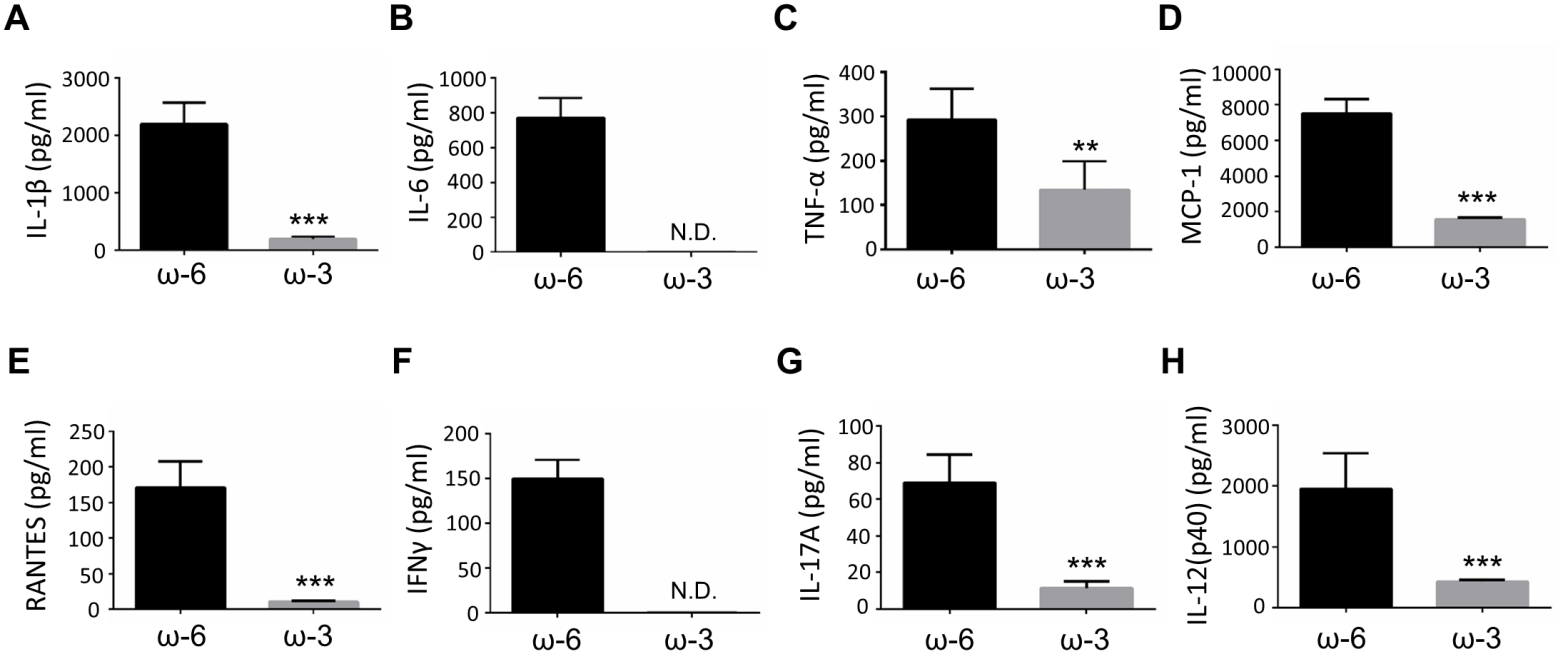
Concentrations of inflammatory cytokines and chemokines in intraocular fluid of EAU mice. The concentrations of IL-1β (A), IL-6 (B), TNF-α (C), MCP-1 (D), RANTES (E), IFN-γ (F), IL-17A (G), and IL-12(p40) (H) in intraocular fluid of EAU mice were determined with a multiplex assay at 14 days after injection of hIRBP(1–20) and maintenance on an ω-6 or ω-3 LCPUFA diet. Data are means ± SD (*n* = 10 for both ω-6 and ω-3 groups). ***P < 0.001, **P < 0.01 versus ω-6 mice. ND, not detected.

To investigate the mechanism by which the dietary intake of ω-3 LCPUFAs suppresses inflammation in EAU mice, we measured the concentrations of inflammatory mediators in intraocular fluid at 14 days after EAU induction with the use of a multiplex assay system (S1 Fig). The concentrations of the inflammatory cytokines interleukin (IL)–1β, IL-6, and tumor necrosis factor–α (TNF-α); the chemokines MCP-1 (monocyte chemoattractant protein–1) and RANTES (regulated on activation, normal T expressed and secreted); the Th1 cytokine interferon (IFN)–γ; the Th17 cytokine IL-17A; and the antigen presenting cell (APC)–associated cytokine IL-12 were markedly reduced in the ω-3 group compared with the ω-6 group (Fig 4).

### Abundance of T-bet and RORγt mRNAs in the retina

**Fig 5:**
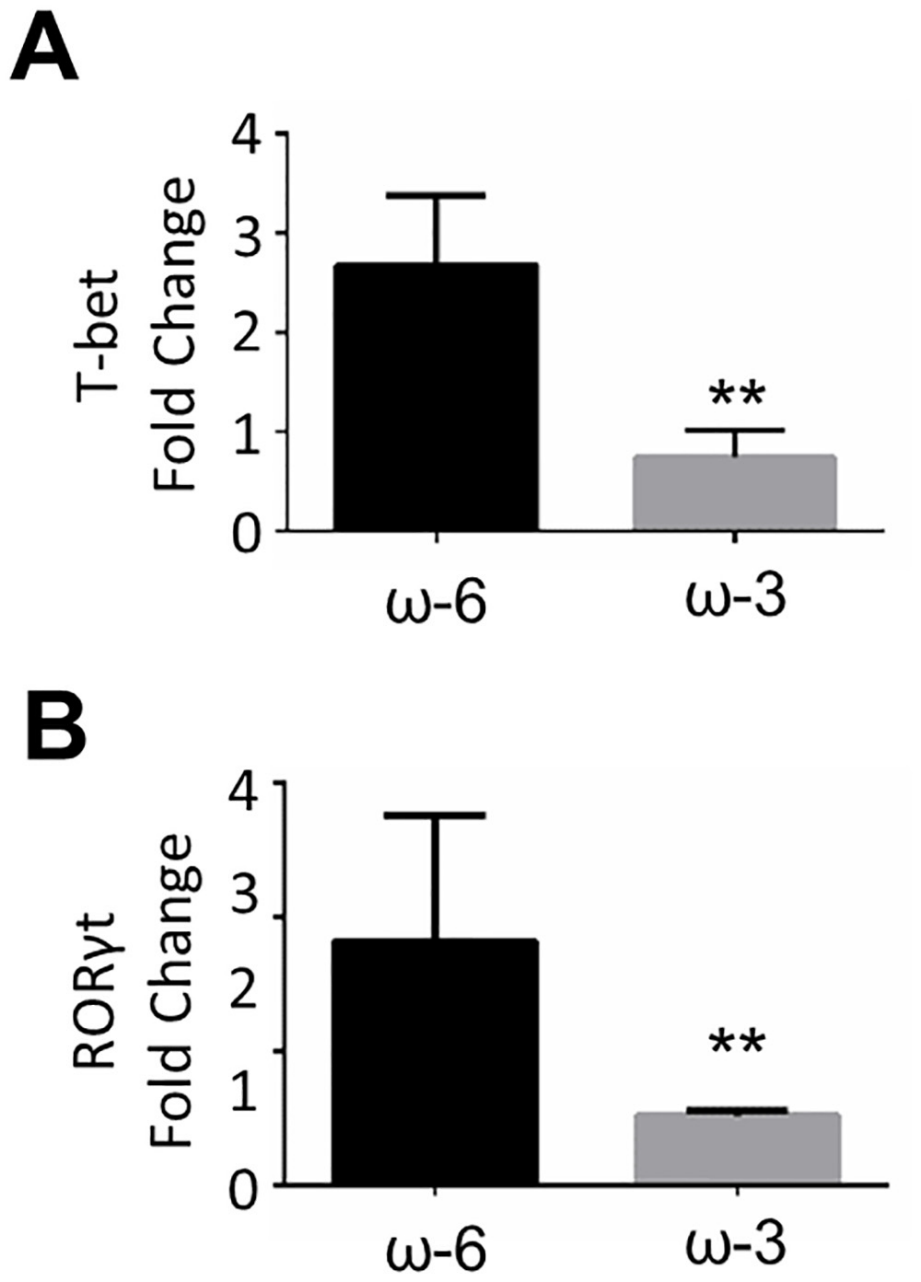
Abundance of T-bet and RORγt mRNAs in the retina of EAU mice. The amounts of T-bet (A) and RORγt (B) mRNAs in the retina of EAU mice were measured by RT and real-time PCR analysis at 21 days after injection of hIRBP(1–20) and maintenance on an ω-6 or ω-3 LCPUFA diet. Data are means ± SD (*n* = 2 for both ω-6 and ω-3 groups). **P < 0.01.

We next examined the abundance of mRNAs for the Th1- and Th17-related transcription factors T-bet and RORγt, respectively, in the retina at 21 days after the induction of EAU. Quantitative RT-PCR analysis revealed that the amounts of both T-bet (Fig 5A) and RORγt (Fig 5B) mRNAs were significantly reduced in the ω-3 group compared with the ω-6 group. These results suggested that ω-3 LCPUFAs suppress intraocular inflammation in part by inhibiting the production of inflammatory mediators by Th1 and Th17 cells in the retina.

### Production of IFN-γ and IL-17 by CD4^+^ T cells

**Fig 6:**
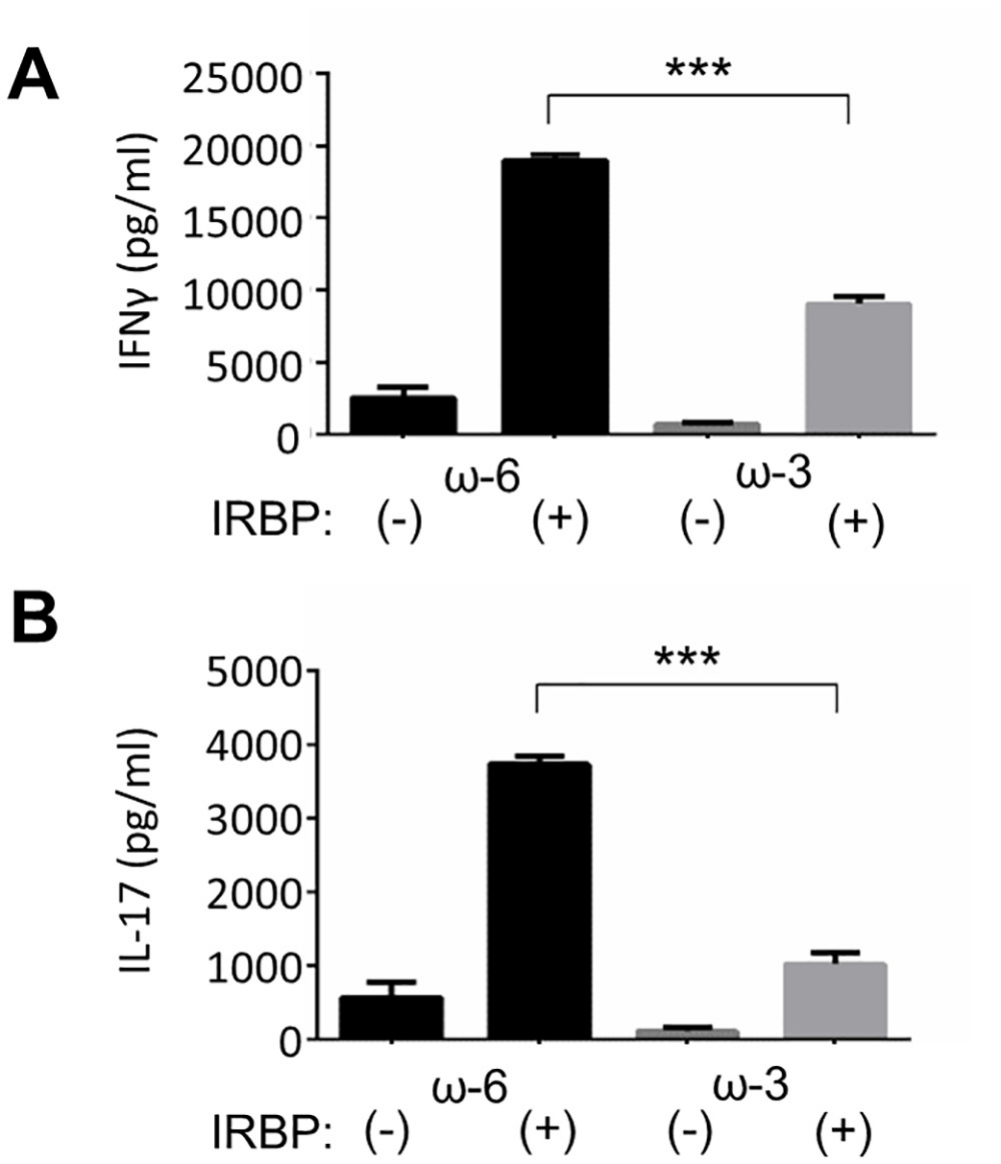
Production of IFN-γ and IL-17 by CD4^+^ T cells isolated from EAU mice. CD4^+^ T cells isolated from lymph nodes of EAU mice at 14 days after injection of hIRBP(1–20) and maintenance on an ω-6 or ω-3 LCPUFA diet were incubated in the absence or presence of hIRBP(1–20) for 48 h, after which the concentrations of IFN-γ (A) and IL-17 (B) in the culture supernatants were measured with ELISAs. The cytokine concentrations for the stimulated cells are presented as means ± SD (*n* = 4 for both ω-6 and ω-3 groups). ***P < 0.001.

The production of inflammatory cytokines by CD4^+^ T cells isolated from the lymph nodes of mice at 14 days after EAU induction was measured with the use of ELISAs. The production of IFN-γ (Fig 6A) and IL-17 (Fig 6B) by CD4^+^ T cells stimulated with hIRBP(1–20) in vitro was significantly reduced for mice in the ω-3 group compared with those in the ω-6 group.

### Abundance of T-bet and RORγt mRNAs in CD4^+^ T cells

We also examined the abundance of mRNAs for T-bet and RORγt in CD4^+^ T cells isolated from the lymph nodes of mice at 21 days after EAU induction. The amounts of all both mRNAs in cells stimulated with hIRBP(1–20) in vitro were markedly reduced for mice in the ω-3 group compared with those in the ω-6 group (S2 Fig).

### Serum lipid profile

We analyzed the lipid profile of serum collected from mice at 14 days after EAU induction and maintenance on control, ω-6 LCPUFA, or ω-3-LCPUFA diets (Table 1). The concentrations of total saturated fatty acids did not differ substantially among the three groups. The concentrations of the principal ω-3 LCPUFAs (EPA and DHA) and total ω-3 LCPUFAs as well as the ω-3/ω-6 LCPUFA ratio were increased in mice fed the ω-3 LCPUFA diet compared with those fed the ω-6 LCPUFA or control diet. Conversely, the concentrations of AA and total ω-6 LCPUFAs were reduced in mice fed the ω-3 LCPUFA diet compared with those fed the ω-6 LCPUFA diet.

**Table 1:** Fatty acid concentrations (ng/ml) in serum of EAU mice.

Fatty acid	Control diet	ω-6 LCPUFA diet	ω-3 LCPUFA diet
***Saturated***			
PA (16:0)	314.6 (19.96)	337.7 (19.42)	275.5 (18.11)
SA (18:0)	163.9 (10.40)	189.8 (10.91)	165.7 (10.89)
Total	495.1 (21.42)	543.9 (31.27)	455.8 (29.97)
***ω-6 LCPUFAs***			
LA (18:2ω6)	454.7 (28.84)	411.0 (23.46)	326.1 (21.44)
AA (20:4ω6)	129.8 (8.23)	348.9 (20.06)	117.8 (7.75)
DTA (22:4ω6)	2.9 (0.18)	9.8 (0.56)	<1.0 (<0.01)
Total	612.8 (38.86)	800.8 (45.86)	463.3 (30.41)
***ω-3 LCPUFAs***			
ALA (18:3ω3)	9.3 (0.59)	0.4 (0.02)	0.7 (0.05)
EPA (20:5ω3)	49.1 (3.11)	0.6 (0.03)	145.1 (9.54)
DHA (22:6ω3)	122.7 (7.78)	68.9 (3.96)	254.9 (16.76)
Total	188.6 (11.96)	70.9 (4.02)	416 (27.36)
**ω-3/ω-6 ratio**	0.308	0.089	0.898
**EPA/AA ratio**	0.38	0.002	1.23

Fatty acid concentrations in serum of EAU mice were measured at 14 days after injection of hIRBP(1–20) and maintenance on a control, ω-6 LCPUFA, or ω-3 LCPUFA diet. Values in parentheses represent the percentage of total fatty acids by weight. Data are means (*n* = 5). PA, palmitic acid; SA, stearic acid; LA, linoleic acid; AA, arachidonic acid; DTA, docosatetraenoic acid; ALA, α-linolenic acid; EPA, eicosapentaenoic acid; DHA, docosahexaenoic acid.

## Discussion

We have shown that dietary ω-3 LCPUFAs suppressed intraocular inflammation, as evaluated both clinically and histologically, in mice with EAU. Similar effects were not observed in mice fed a diet supplemented with ω-6 LCPUFAs. Dietary ω-3 LCPUFAs reduced the concentrations of inflammatory cytokines and chemokines in intraocular fluid of EAU mice as well as attenuated the expression of key transcription factors in retinal Th1 and Th17 cells. Furthermore, the expression of these transcription factors as well as the production of IFN-γ and IL-17 were down-regulated in lymph node cells of EAU mice fed ω-3 LCPUFAs. Our results thus indicate that ω-3 LCPUFAs ameliorate EAU by inhibiting both systemic and retinal Th1 and Th17 cells.

Dietary enrichment with ω-3 LCPUFAs such as DHA and EPA present in fish oil and certain plant and nut oils [4,25,26] has been shown to protect against inflammation [27–29], pathological angiogenesis [3], and tumorigenesis [30]. ω-3 LCPUFAs have also been found to suppress ocular inflammation, such as that associated with dry eye [31] and EIU [10], as well as ocular neovascularization such as that associated with age-related macular degeneration [3,11,12]. Oral administration of EPA thus reduced the extent of leukocyte adhesion to retinal vessels as well as the production of IL-6 and MCP-1 in EIU mice [10]. We have now shown that dietary ω-3 LCPUFAs reduced the concentrations of various pro-inflammatory cytokines and chemokines, including IL-6 and MCP-1, in intraocular fluid of EAU mice compared with those present in animals fed a diet enriched in ω-6 LCPUFAs. We focused our analysis, however, on the role of Th1 and Th17 cells in EAU, finding that ω-3 LCPUFAs suppress EAU at least in part by inhibiting the differentiation and inflammatory responses of systemic and local Th1 and Th17 cells.

Th17 cells and IL-17 contribute to intraocular inflammation in animal models of uveitis [24]. In humans, the number of Th17 cells increases during acute uveitis and then decreases after treatment [32–34]. Th17 cell lines established from a patient with Behçet’s uveitis were found to express high levels of the Th17 cell—related transcription factor RORγt, and this expression was attenuated by exposure of the cells to infliximab, a monoclonal antibody specific for TNF-α [35]. Dietary ω-3 LCPUFAs were shown to reduce the accumulation of Th1 and Th17 cells as well as inflammatory macrophage subsets in experimental colitis [36,37]. We have now shown that dietary ω-3 LCPUFAs reduced the concentrations of Th1 and Th17 cytokines (IFN-γ and IL-17, respectively) in intraocular fluid of EAU mice as well as attenuated the production of these cytokines by CD4^+^ T cells isolated from lymph nodes of these animals. Furthermore, ω-3 LCPUFAs down-regulated the expression of the Th1 and Th17 transcription factors T-bet and RORγt, respectively, in both the retina and CD4^+^ T cells isolated from lymph nodes of EAU mice. Our results thus indicate that ω-3 LCPUFAs inhibit Th1 and Th17 cell differentiation and thereby suppress the induction of EAU.

We did not examine the effects of dietary intake of ω-3 LCPUFAs on APCs such as macrophages and B cells in the present study, although we did find that the concentration of the APC-associated cytokine IL-12 was reduced in intraocular fluid of EAU mice fed ω-3 LCPUFAs compared with those fed ω-6 LCPUFAs. Given that ocular inflammation in EAU is dependent on APCs as well as on T cells [38,39]—with macrophages, for example, having been found to contribute to tissue damage—the effects of ω-3 LCPUFAs on these cells in EAU warrant further investigation [40,41].

Several clinical studies have shown that ω-3 LCPUFAs reduce inflammation and may help lower the risk of chronic diseases such as heart disease, cancer, and rheumatoid arthritis [9,42,43]. These clinical studies have evaluated not only the beneficial effects but also the safety of ω-3 LCPUFA supplementation, with ω-3 LCPUFAs derived from fish oil and certain plants having the advantage in this regard of being natural products. Our findings now suggest that ω-3 LCPUFAs warrant further investigation as a potential treatment for uveitis patients. Further progress in the clinical settings in uveitis will likely provide a basis for the development of new treatments for uveitis in the future.

## Supporting Information

S1 Fig Concentrations of inflammatory cytokines and chemokines in intraocular fluid of EAU mice. The concentrations of 23 cytokines and chemokines in intraocular fluid of mice at 14 days after injection of hIRBP(1–20) and maintenance on an ω-6 or ω-3 LCPUFA diet were determined with a multiplex assay. Data are means ± SD of triplicate determinations for analysis of representative animals (*n* = 7). Inverted triangle, not detected.
(TIF)

S2 Fig Abundance of T-bet and RORγt mRNAs in CD4^+^ T cells isolated from EAU mice. CD4^+^ T cells isolated from lymph nodes of EAU mice at 21 days after injection of hIRBP(1–20) and maintenance on an ω-6 or ω-3 LCPUFA diet the amounts of T-bet (A) and RORγt (B) mRNAs were measured by RT and real-time PCR analysis. The mRNA amounts for stimulated cells were corrected for those for the nonstimulated cells and are presented as measured values. ND, not detected.
(TIF)
