# Supplementary figures and images for: Dietary Omega-3 Fatty Acids Suppress Experimental Autoimmune Uveitis in Association with Inhibition of Th1 and Th17 Cell Function
Source: PLoS One. 2015 Sep 22;10(9):e0138241. doi: 10.1371/journal.pone.0138241 (PMC4578775; doi:10.1371/journal.pone.0138241)

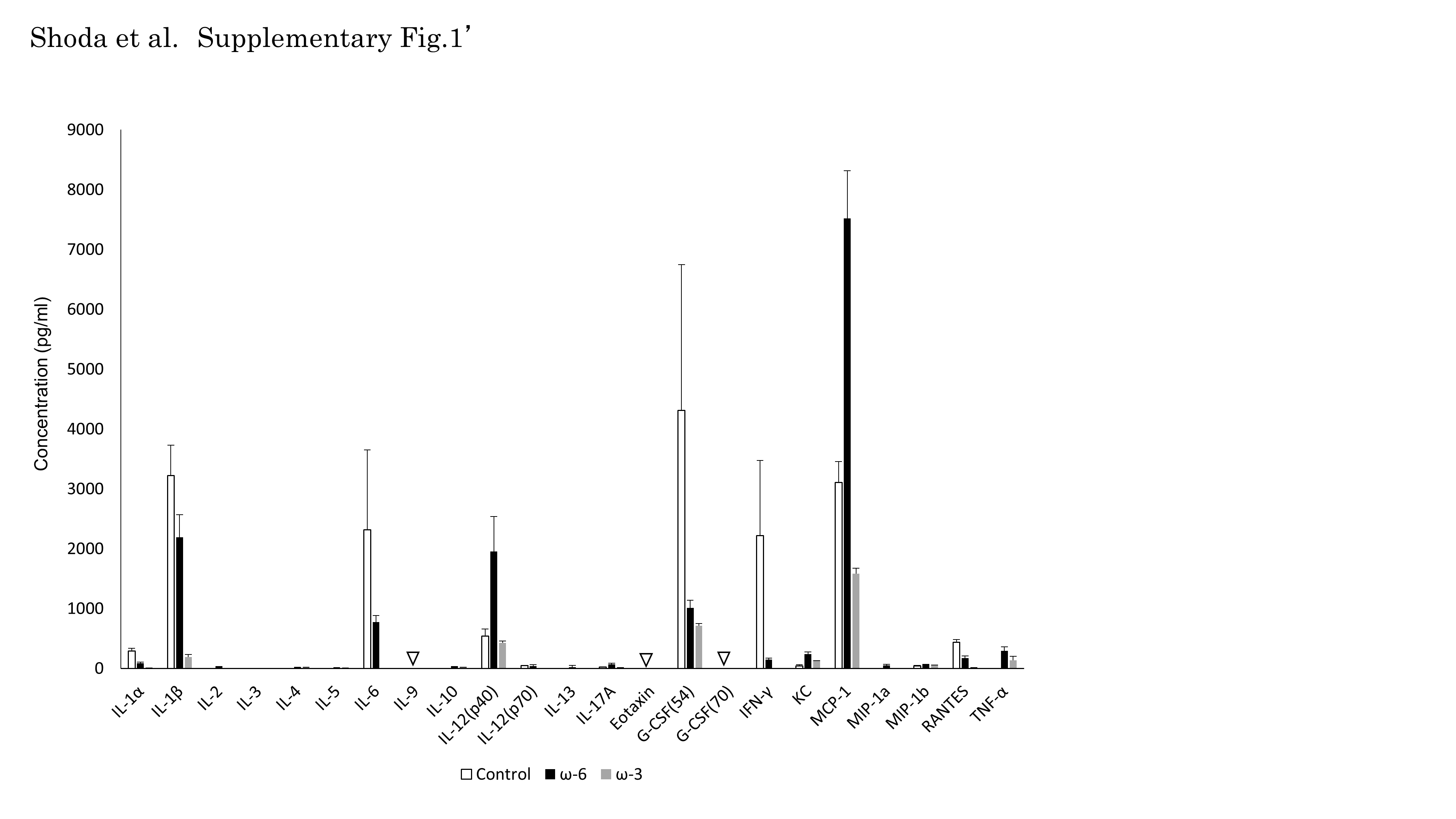

Supplement: S1 Fig — The concentrations of 23 cytokines and chemokines in intraocular fluid of mice at 14 days after injection of hIRBP(1–20) and maintenance on an ω-6 or ω-3 LCPUFA diet were determined with a multiplex assay. Data are means ± SD of triplicate determinations for analysis of representative animals (n = 7). Inverted triangle, not detected. (TIF) [file pone.0138241.s001.tif]

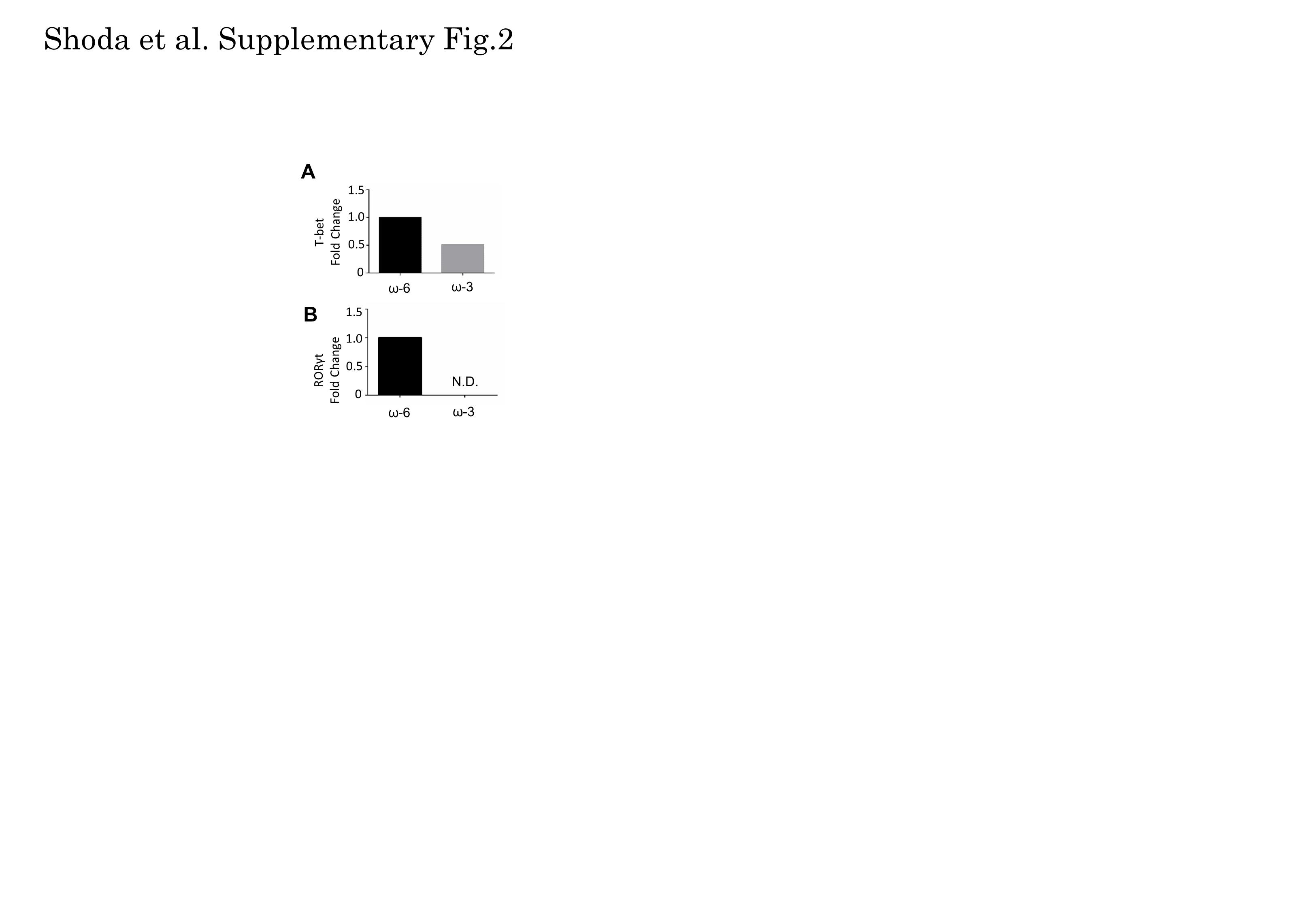

Supplement: S2 Fig — CD4+ T cells isolated from lymph nodes of EAU mice at 21 days after injection of hIRBP(1–20) and maintenance on an ω-6 or ω-3 LCPUFA diet the amounts of T-bet (A) and RORγt (B) mRNAs were measured by RT and real-time PCR analysis. The mRNA amounts for stimulated cells were corrected for those for the nonstimulated cells and are presented as measured values. ND, not detected. (TIF) [file pone.0138241.s002.tif]
